# Manipulating player numbers in small-sided soccer games: effects on numerical superiority and inferiority across physiological, physical, technical, and tactical performance

**DOI:** 10.3389/fspor.2026.1813770

**Published:** 2026-04-15

**Authors:** Michael C. Rumpf, Johannes Jäger, Rhodri S. Lloyd, Matthias Lochmann

**Affiliations:** 1 Department of Sport Science and Sport, Friedrich-Alexander University Erlangen-Nürnberg, Erlangen, Germany; 2 Footballscience.net, Dreieich, Germany; 3 Sport Performance Research Institute New Zealand, AUT-University, Auckland, New Zealand; 4 Youth Physical Development Center, Cardiff School of Sport and Health Sciences, Cardiff Metropolitan University, Cardiff, United Kingdom; 5 Center for Sport Science and Human Performance, Waikato Institute of Technology, Hamilton, New Zealand

**Keywords:** football, performance analysis, player number, small-sided games, task constraint

## Abstract

Small-sided games (SSG) are widely used in soccer training to replicate the physiological, physical, technical, and tactical demands of competition within representative contexts. Coaches often manipulate task constraints by creating numerical superiority or inferiority, which imposes specific demands and elicits targeted adaptations. This review aimed to synthesize the effects of numerical imbalances on player performance across physiological, physical, technical, and tactical domains. Evidence indicates that playing in numerical superiority consistently reduces physiological intensity (e.g., mean heart rate, time >90% HRmax) and high-intensity physical load (e.g., total distance, sprinting, accelerations), while increasing low-intensity activity such as walking. Oppositely, technical performance improves, with a higher number of passes and overall actions. Tactical behavior also adapts, with teams expanding playing length and width, covering more space near the opposition goal, and increasing attacking opportunities, while exploratory behavior and proximity to their own goal decrease. Conversely, teams in numerical inferiority adopt compensatory strategies, focusing on defensive organization and goal protection, which can reduce physical output and exploratory movement, particularly under high-inferiority conditions. Numerical imbalances in SSG seem to modulate physiological load, physical activity, technical execution, and tactical behavior. These findings provide a framework for optimizing training design and session planning in soccer, enabling coaches to manipulate player numbers to target specific performance outcomes.

## Introduction

A major principle of invasion team sports involves outnumbering the opposition (creation of numerical overloads) during different performance phases (attack and defense) (1, 2). By exploiting numerical superiority, teams might create spatial and temporal advantages that increase the probability of successful offensive actions and disrupt the opponent's defensive organization (2). Conversely, teams experiencing numerical inferiority must adopt compensatory tactical behaviors, which may increase defensive compactness and prioritization of defensive positioning (3).

In soccer, numerical imbalances can arise at various moments during a match. They often arise during counterattacks following turnovers (4), when players transition from defense to offense, or through disciplinary measures, such as red cards, which reduce the number of players on a team. Player dismissals have substantial tactical and physical consequences for both the team reduced in numbers and the team with numerical superiority (5). For example, teams reduced to 10 players typically spend more time in their defensive third and less in their attacking third, directly affecting scoring opportunities (5–7). Moreover, these situations also influence physical demands. Studies have shown that both total distance (TD) covered per minute and high-speed running distance per minute decrease after a dismissal, reflecting adaptive pacing strategies and changes in team dynamics (5).

Recognizing the practical implications of numerical imbalances, coaches frequently incorporate controlled scenarios of numerical inferiority or superiority during small-sided games (SSG) to replicate game-like situations in a representative manner, fostering tactical adaptability, while simultaneously influencing players’ physiological, physical, and technical capacities (3, 8–16).

By manipulating player numbers (e.g., 5 vs. 4, 5 vs. 3) coaches can elicit targeted physiological, technical, tactical, and perceptual responses. The application of numerical imbalances in SSG has been shown to influence multiple performance indicators. Physiologically, such manipulations alter heart rate (HR) responses, blood lactate accumulation, and metabolic load (8, 17), Physically, they affect running in distances covered at various speeds (17). Technically, differences have been observed in dribbling and receptions (8). Tactically, SSG with numerical manipulations promote adaptations in team coordination and interpersonal-distances (3, 10, 17–19), Additionally, perceptual measures such as ratings of perceived exertion (RPE) reflect the altered psychophysiological demands imposed by changes in player ratios (20, 21).

Despite the extensive use of numerical imbalances in SSG and the growing number of studies examining their effects, there remains a lack of comprehensive synthesis integrating findings across physiological, physical, technical, and tactical outcomes. A nuanced understanding of how numerical superiority and inferiority influence player performance is essential for informing training design and optimizing session planning according to existing literature. Therefore, this narrative mini-review aims to provide the first integrated synthesis of the scientific evidence regarding the effects of numerical imbalances on player performance indicators in SSG. By consolidating current knowledge, this review seeks to highlight the multifaceted impact of numerical manipulations and offer practical insights to guide both future research and the application of SSG in soccer coaching contexts.

## Methods

Although formal literature search procedures are not mandatory for narrative reviews, their inclusion can help mitigate the elevated risk of selection and inclusion bias associated with this review format (22), particularly when the number of included references is limited (23). In the present mini-review, a pragmatic search strategy was therefore implemented to enhance reproducibility, while a fully systematic review methodology was not adopted due to the exploratory and integrative aim of summarizing emerging evidence across heterogeneous study design (24). This approach enabled the inclusion of a broad range of relevant literature without the restrictive criteria required for systematic reviews, thereby facilitating a comprehensive and interpretative synthesis of the current evidence base (25).

A basic search strategy, including predefined eligibility criteria, was applied. Major databases (PubMed, Web of Science, SPORTDiscus, Scopus, and Google Scholar) were searched from January 2000 to December 2025 using combinations of the following keywords: “coaching”, “training”, “soccer”, “football”, “small-sided games”, “players”, “overload”, “numerical inferiority”, “numerical superiority”, and “numerical imbalance”. The search was supplemented by screening grey literature (https://opengrey.eu), examining the reference lists of all included studies, and reviewing previous reviews on related topics. Inclusion criteria required studies to (1) examine numerical superiority or inferiority in soccer SSGs and (2) report effects on physiological, physical, technical, or tactical outcomes. Studies were excluded if they were (1) not published in English, (2) unrelated to soccer SSGs or numerical imbalances, or (3) lacked original data or relevant outcome measures. Identification of suitable articles followed a two-stage process consisting of (i) title and abstract screening and (ii) full-text assessment of potentially relevant studies. From the included studies, data were extracted on participant characteristics, small-sided game formats, training protocols, and outcome variables.

In line with the narrative scope of this mini-review, no formal risk-of-bias or methodological quality assessment was conducted. The primary objective was to provide a concise synthesis of the current literature to offer a broad and accessible overview of the research topic, rather than to produce an evidence-graded evaluation (25).

## Results

A total of 135 records were identified through database searches. After the removal of duplicates, 110 records were screened based on titles and abstracts, of which 70 were excluded. The remaining 40 reports were retrieved and assessed for eligibility. Of these, 22 were excluded due to the absence of a numerical imbalance (*n* = 16), the reporting of irrelevant or incorrect outcomes (*n* = 5), or the lack of SSG (*n* = 1). Ultimately, 18 studies met the inclusion criteria and were included in the review.

### Playing in numerical superiority in SSG

The included studies used varying terminology to describe numerically imbalanced SSG, referring to such formats as unbalanced (26), overloaded (27) and underloaded (1). In several cases, the degree of imbalance was further categorized according to the magnitude of numerical inferiority or superiority. Specifically, scenarios were categorized as low inferiority- (−1 players; e.g., 3 vs. 4 or 4 vs. 5) and high-inferiority (≤ −2 players; e.g., 2 vs. 4 or 4 vs. 7) (14, 19, 28). Furthermore, conditions were described as low superiority (+1 players; e.g., 4 vs. 3 or 6 vs. 5) or high-superiority (≥ + 2 players; e.g., 4 vs. 2 or 7 vs. 4) (28). Across game formats, these classifications were maintained such that teams in inferiority and superiority participated in both offensive and defensive phases of play.

Across the 18 included studies, ten investigated small-sided games played in numerical superiority, synthesizing findings from a total of 176 players (1, 12, 14, 17, 18, 21, 26–29). These studies were conducted in both youth (1, 12, 26, 27, 29) and adult (14, 17, 18, 21, 28) populations (Table 1). The studies examined a range of formats, including 2 vs. 4 (28), 3 vs. 4 (12, 14, 18, 21, 28, 29), 3 vs. 5 (1), 4 vs. 5 (1, 14, 17, 18, 21, 28), 4 vs. 6 (26–28), 4 vs. 7 (14, 18, 21), and 5 vs. 6 (29). A summary of all included studies playing in numerical superiority in SSG and their main outcomes are presented in Table 1.

**Table 1:** All reference regarding playing in numerical superiority on outcome variables.

Reference	Population	Playing format	Set-up protocol	Outcome in variable as per condition
Silva et al. 2014 (1)	*N* = 20, male youth 17.6 years of age, U19 national and regional level players	5 vs. 5 5 vs. 4 5 vs. 3	**Pitch size:** 47.3 × 30.6 m **Protocol:** 6 min with 6 min passive recovery	**Tactical variables:** Stretch index ↑, centroids distance to mid-goal ↓
Garcia-Angulo et al. 2024 (12)	*N* = 8, male youth 10.3 years of age, U11 regional level players	4 vs. 4 4 vs. 3	**Pitch size:** 40 × 40 m, 35 × 40 m **Protocol:** 1 × 10 min	**Technical variables:** # of actions ↑ **Tactical variables:** Interpersonal Dist ↑
Goncalves et al. 2016 (14)	*N* = 22, male adult 19.6 years of age, professional players from 2nd Spanish division *N* = 22, male adult 23.1 years of age, amateur players from undergraduate sport science course	4 vs. 3 4 vs. 5 4 vs. 7	**Pitch size:** 40 × 30 m **Protocol:** 3 min with 4 min passive recovery	**Tactical variables:** Dist to own centroid ↑, Dist to opponent centroid ↑, Dist to nearest opponent ↑
Sampaio et al. 2014 (17)	*N* = 24, male adult 20.8 years of age, players trained 4 times/week	5 vs. 4 4 vs. 5	**Pitch size:** 60 × 40 m **Protocol:** 7 × 5 min with 3 min passive recovery	**Physiological variables:** Time spent >90% HRMax ↓, time spent <75%HRmax ↑, time spent 75–85%HRmax ↑ **Physical variables:** Dist running in speed 16–17.9 km/h ↓ **Tactical variables:** Dist to own centroid ↑, randomness in Dist to team centroid ↑
Torrents et al. 2016 (18)	*N* = 22, male adult 25.6 years of age, professional players *N* = 22, male adult 23.1 years of age, amateur players	4 vs. 3 4 vs. 5 4 vs. 7	**Pitch size:** 40 × 30 m **Protocol:** 3 min with 4 min passive recovery	**Technical variables:** # of passes ↑ **Tactical variables:** Exploratory behavior ↓, # of attacking situations ↑
Torres-Ronda et al. 2015 (21)	*N* = 22, male adult 25.6 years of age, professional players *N* = 22, male adult 23.1 years of age, amateur players	4 vs. 3 4 vs. 5 4 vs. 7	**Pitch size:** 40 × 30 m **Protocol:** 2 × 3 min with 4 min passive recovery	**Physiological variables:** Modified TRIMP ↓, RPE ↓ **Physical variables:** TD ↓, exertion index (indication of accumulative load) ↓, body load ↓
Hill-Haas et al. 2010 (29)	*N* = 16, male youth 15.6 years of age, state team standard	3 vs. 4 3 vs. 3 + 1 5 vs. 6 5 vs. 5 + 1	**Pitch size:** 37 × 28 m; 47 × 35 m **Protocol:** 24 min	**Physiological variables:** RPE ↓
Guard et al. 2021 (26)	*N* = 10, male youth 18.0 years of age, Scottish and UEFA Youth league players	a) 5 vs. 5 b) 6 vs. 4	**Pitch size:** a) 45 × 34, 39 × 39 m, b) 23 × 23 m **Protocol:** a) 4 × 4 min with 3 min passive recovery, b) 8 × 2 min with 1 min passive recovery	**Physiological variables:** %HRmax ↓, time spent >90%HRmax ↓, RPE ↓ **Physical variables:** TD ↓, relative TD ↓, mean velocity ↓, peak velocity ↓, #acc ↓, Dist acc >3 m/s/s ↓, low acc (0–5,8 m/s/s) ↑, player load slow (sum of activity < 2 m/s/s) ↑
Guard et al. 2022 (27)	*N* = 12, male youth 18.0 years of age, Scottish and UEFA Youth league players	a) 6 vs. 6 b) 6 vs. 4	**Pitch size:** a small) 40 × 30 m, a medium) 45 × 34 m, a large) 49 × 37 m; b) 23 × 23 m **Protocol:** a) 4 × 4 min with 2 min passive recovery, b) 8 × 2 min with 1.5 min passive recovery	**Physiological variables:** %HRmax ↓, RPE ↓ **Physical variables:** Relative TD ↓, mean velocity ↓, peak velocity ↓, #acc >3 m/s/s ↓, Dist acc >3 m/s/s ↓, # and Dist of dec < 2.78 m/s/s ↓, player load ↓, work-to-rest ratio ↓
Nunes et al. 2020 (28)	*N* = 20, male adult 22.3 years of age, university-level playing at semi-professional level	4 vs. 2 4 vs. 3 4 vs. 4 4 vs. 5 4 vs. 6	**Pitch size:** 30 × 25 m **Protocol:** 4 × 2 min with 4 min passive recovery	**Physiological variables:** RPE ↓ **Physical variables:** Peak velocity ↓, Dist sprinting >18 km/h ↓, Dist running 9–18 km/h ↓, Dist walking <9.0 km/h ↑ **Technical variables:** # of passes ↑

RPE, rating of perceived exertion; %, percentage; max, maximum; Ave, average; NA, neutral attacker; ND, neutral defender; OF, outside floater; IF, inside floater; GK, goalkeeper; NP, not provided; HR, heart rate; Dist, distance; #, number; Acc, acceleration; Dec, deceleration; ↑, significant increase; ↓, significant decrease.

### Physiological variables

Six studies (*n* = 104 players) indicated that players in numerical superiority generally exhibited lower physiological load compared with numerical inferiority. Youth (26, 27) and adult players (17, 21) demonstrated reduced mean heart rate (%HRmax), less time spent >90% HRmax, lower modified training impulse (TRIMP), and decreased RPE. Simultaneously, low-intensity indicators, such as time spent <75% HRmax or 75%–85% HRmax, increased (17).

Exceptions were limited; one study found no significant differences in %HRmax or blood lactate between superiority and inferiority in youth (29), though a trend toward greater strain during inferiority was noted. The latter was also shown to decrease modified in professional players, with authors concluding that the high-inferiority (4 vs. 7) resulted in strategic tactical behavior focusing on protecting the space around the goal (21).

### Physical variables

Playing in numerical superiority generally reduced physical load, but the magnitude and pattern of these reductions differed between youth and adult players. Across five studies (*n* = 88 players), adult players (17, 21, 28) demonstrated pronounced decreases in total and relative distance covered, mean and peak velocity, sprinting distance 9–18 km/h, >18 km/h, accelerations and decelerations, player load, body load, exertion index, and work-to-rest ratio during numerical superiority. Youth players (26, 27) indicated significant reductions in variables associated with greater physical activity, including total and relative running distance, mean and peak velocity, number of accelerations, number and distance of accelerations >3 m/s/s, number and distance of deceleration < 2.78 m/s/s, player load, and work-to-rest ratio were significantly lower in players in numerical superiority. Simultaneously, both youth and adult players increased activities associated with lower intensity, such as walking distance (<9.0 km/h), low-acceleration distance (0–5,8 m/s/s), and player load slow (sum of activity < 2 m/s/s) (26, 28). Notably, extreme high-inferiority (−3 players or more) reversed some of these trends, particularly in adults (21). In such scenarios, physical output was reduced due to strategic behavior, with teams prioritizing goal protection and controlling the surrounding space resulting in lower physical outputs (21).

### Technical variables

Three studies (*n* = 50 players) including youth and adult populations found that superiority was associated with a higher number of passes and overall technical actions (sum of passes, shots, lost balls, and other actions) (12, 18, 28). However, given the limited number of studies reporting these variables, these results should be interpreted with caution. Data on numerical inferiority are scarce, but available evidence suggests fewer technical opportunities due to increased defensive pressure.

### Tactical variables

Numerical superiority generally led to greater spatial coverage and flexibility. In slight superiority scenarios (+1 player), youth players showed increases in stretch index and reduced distance to mid-goal, reflecting improved spacing and more effective offensive positioning (1). Interpersonal distance also increased in slight superiority (+1/2), providing more time and space for technical actions (12). With higher superiority (+2/+3 players), adult players exhibited larger tactical adaptations, including increased distances to own and opponent centroids and enhanced (14, 17), and exploratory behavior (18).

Combined with technical improvements, high superiority (>+2 players) led to more attacking situations and greater tactical diversity (18). Conversely, numerical inferiority constrained tactical options, with high-inferiority scenarios encouraging compact defensive positioning and limiting offensive flexibility (19). Slight inferiority (−1 player) may encourage individual tactical actions and decision-making (28).

### Fixed superiority vs. even player number SSG

Unlike the previous section, which focused on comparisons between numerical superiority and inferiority, the following section examines differences between small-sided games played with a fixed numerical superiority or inferiority and games played with an equal number of players. Eight studies (*n* = 138 players) compared fixed numerical superiority/inferiority with even-numbered formats (3, 8, 9, 11, 13, 19, 20, 30), predominantly from youth populations (8, 9, 11, 13, 20, 30), with only two studies conducted in adult players (3, 19) populations.

In youth players, superiority reduced physiological load such as HRave (8, 30), HRmax (30), blood lactate (8) and RPE (20, 30). Evidence regarding physical responses to fixed numerical superiority versus evenly numbered formats is limited and restricted to youth players. Praca et al. (30) registered significantly lower TD, distance covered in speed 14.4–21.5 km/h and higher distance covered in 0–7.2 km/h in superiority games. The absence of adult data precludes firm conclusions regarding performance-level differences in physical responses. Regarding technical measures, only one study, conducted in youth players, reported significantly higher numbers of dribbling actions during numerical superiority compared with even-numbered formats (11).

Tactical outcomes were investigated more extensively across both youth and adult populations. In youth players, superiority increased spatial dispersion (playing length, width, length-to-width ratio) in some studies (13), while others reported no significant differences (9). Adult players showed more nuanced tactical adaptations, such as reduced team surface area and closer distances to geometric centers during superiority (3) alongside increased tactical diversity and flexibility (19). A summary of all included studies playing in fixed superiority in SSG and their main outcomes are presented in Table 2.

**Table 2:** All reference regarding fixed numerical superiority vs. even player number SSG on outcome variables.

Reference	Population	Playing format	Set-up protocol	Outcome in variable as per condition
Rochael & Praca 2023 (9)	*N* = 40, male youth 16.4 years of age, U17 elite youth academies	4 vs. 4 5 vs. 4	**Pitch size:** 41 × 29 m **Protocol:** 4 × 4 min with 4 min passive recovery	*4 vs. 4* **Tactical variables:** Spatial exploration index ↑
Sousa et al. 2021 (20)	*N* = 10, male youth 13.6 years of age, federated players training 3 times/week	4 vs. 4 4 vs. 5 4 vs. 6	**Pitch size:** 40 × 30 m, 35 × 25 m **Protocol:** 6 × 5 min with 5 min passive recovery	*4 vs. 4 compared to 4 vs. 6/ 4 vs. 5* **Physiological variables:** RPE ↑ (on small pitch size), RPE ↑ (on small pitch size)
Praxedes et al. 2016 (11)	*N* = 20, male youth 10.6 years of age	3 vs. 3 3 vs. 2	**Pitch size:** 35 × 20 m **Protocol:** 2 × 4 min with 1 min passive recovery	*3 vs. 2* **Technical variables:** # of dribblings ↑
Travassos et al. 2014 (3)	*N* = 15, male adult 19.6 years of age, intermediate level playing at an associate level	4 vs. 4 4 vs. 3	**Pitch size:** 40 × 20 m **Protocol:** 2 × 5 min	*4 vs. 3* **Tactical variables:** Dist attacker from geometric centre ↓, Dist defender from geometric centre ↓, surface area attacking team ↓, surface area defending team ↓, Dist of geometric centre of attacking – defending team ↑
Ric et al. 2016 (19)	*N* = 8, male adult 26 years of age, professional players training 5 times/week	4 vs. 3 4 vs. 5 4 vs. 7	**Pitch size:** 40 × 30 m **Protocol:** 2 × 3 min with 4 min passive recovery	**Tactical variables:** Tactical diversity ↑ and flexibility ↑
Bekris et al. 2012 (8)	*N* = 9, male youth 17.2 years of age, amateur players training 3 times/week	a) 3 vs. 3 a) 3 vs. 3 + 1 NA a) 3 vs. 3 + 1 ND a) 4 vs. 3 b) 4 vs. 4 b) 4 vs. 4 + 1 NA b) 4 vs. 4 + 1 ND b) 5 vs. 4	**Pitch size:** a) 20 × 25 m, b) 30 × 25 m **Protocol:** 3 min with 12 min passive recovery	*4 vs. 3* **Physiological variables:** HRMean ↓, duration >85% HRMax ↓
Praca et al. 2016 (13)	*N* = 18 male youth 16.4 years of age, from a professional club	3 vs. 3 3 vs. 3 + 2 OF 3 vs. 3 + 1 IF	**Pitch size:** 36 × 27 m **Protocol:** 2 × 4 min with 4 min passive recovery	*3 vs. 3* *±* *1 IF vs. 3vs 3 & 3 vs. 3* *±* *2 OF* **Tactical variables:** Length ↑, width ↑ *3 vs. 3* *±* *2 OF vs. 3vs 3* **Tactical variables:** Tactical variables: Width ↑ *3 vs. 3* *±* *2 OF vs. 3vs 3 & 3 vs. 3* *±* *1 IF* **Tactical variables:** Centroid distance ↑, length-per-width ratio ↑ *3 vs. 3* *±* *1 IF vs. 3vs 3* **Tactical variables:** Length-per-width ratio ↑
Praca et al. 2018 (30)	*N* = 18 male youth 16.4 years of age, from a professional club	3 vs. 3 4 vs. 3	**Pitch size:** 36 × 27 m **Protocol:** 2 × 4 min with 4 min passive recovery	*4 vs. 3* **Physiological variables:** HRMean ↓, HRMax ↓, RPE ↓ **Physical variables:** TD ↓, Dist covered 14.4–21.5 km/h ↓, Dist covered 0–7.2 km/h ↑

RPE, rating of perceived exertion; %, percentage; max, maximum; Ave, average; NA, neutral attacker; ND, neutral defender; OF, outside floater; IF, inside floater; GK, goalkeeper; NP, not provided; HR, heart rate; Dist, distance; #, number; Acc, acceleration; Dec, deceleration; ↑, significant increase; ↓, significant decrease.

## Discussion

This narrative mini review aimed to synthesize current evidence on the effects of numerical superiority and inferiority during SSG in soccer. Overall, playing in numerical superiority is consistently associated with reduced physiological and physical demands. A likely explanation is that the additional player provides more passing options and greater spatial control, reducing the need for frequent high-intensity off-ball movements and urgent defensive actions. Consequently, teams can maintain possession with lower metabolic and neuromuscular strain. Although most studies support this pattern, one study found no significant differences in %HRmax or blood lactate between superiority and inferiority in youth players (29). This discrepancy may reflect methodological differences, including the younger age of participants. It also suggests that youth players may adopt pacing strategies, with physiological responses to numerical manipulations, such as anaerobic energy contribution (31), being less pronounced in younger populations.

Conversely, technical actions tend to increase, while tactical behavior adapts through greater spatial dispersion and expanded playing length and width (32). Reduced opponent pressure and increased interpersonal distances provide players with more time and information to execute technical skills (14), likely facilitating higher passing rates and the execution of more technically demanding actions. These patterns were observed in both youth and adult players, although the overall evidence base remains limited.

Teams in numerical inferiority, by comparison, often adopt compensatory strategies such as reduced exploratory behaviour and a stronger emphasis on defensive organization. This typically results in more compact and centrally oriented structures aimed at minimizing exploitable space and protecting areas close to goal (32). Such adaptations reflect emergent collective behaviours shaped by altered interaction constraints, whereby teams reorganize to maintain functional stability under disadvantage (10).

Comparisons with even-numbered SSG further reinforce that manipulating player numbers reliably modulates performance across physiological, physical, technical, and tactical domains. Importantly, the magnitude of numerical imbalance and the context of the SSG (e.g., age group, player level) influence the direction and extent of these effects. Although youth and adult players show broadly similar trends, adult and higher-level players seem to exploit numerical superiority more efficiently, likely due to greater tactical knowledge and team coordination (33). From an applied perspective, manipulating numerical balance during SSGs represents a tool for coaches to regulate training load and target specific performance outcomes. Numerical superiority can be used to reduce physical demands and physiological stress while increasing opportunities for technical execution and tactical exploration, making it particularly suitable for technical-tactical learning phases or lower-load training sessions, especially in youth soccer. Conversely, tasks with high numerical inferiority may diminish physical stimuli if the task becomes insufficiently challenging. In professional contexts (21), coaches aiming to impose overload, may consider implementing a moderate numerical inferiority of −1 or −2 players.

This review is limited by the relatively small number of available studies and the heterogeneity of player populations and SSG formats. In addition, most studies have been conducted in male players, and only a few have directly compared different competitive levels. Therefore, future research should examine how player age, expertise, and gender interact with numerical manipulations, as these factors are likely to moderate physiological responses, physical demands, and technical-tactical patterns during SSGs.

## Conclusion

This review aimed to summarize the current evidence regarding the effects of manipulating player numbers through numerical superiority and inferiority in SSGs. By synthesizing the available literature, the findings indicate that numerical imbalance acts as a task constraint capable of either increasing or reducing physical load and physiological responses. In general, playing in numerical superiority is associated with reduced physical and physiological demands, while simultaneously facilitating greater technical involvement and promoting tactical adaptations such as increased spatial coverage and flexibility. Conversely, numerical inferiority tends to elevate physical and physiological strain and encourages more compact and defensively oriented tactical behavior.

These outcomes should be interpreted within the context of player age and skill level, as responses to numerical manipulations may differ between youth and adult players as well as between amateur and professional populations. The heterogeneity of study designs and participant characteristics highlights the need for further research to clarify how numerical superiority and inferiority operate across different contexts. Overall, manipulating player numbers represents a practical tool for coaches when designing SSGs to regulate training load and target specific physiological, technical, and tactical performance outcomes in soccer.
